# Construction and validation of a perioperative blood transfusion model for patients undergoing total hip arthroplasty with osteonecrosis of the femoral head based on machine learning

**DOI:** 10.3389/fmed.2025.1471746

**Published:** 2025-09-11

**Authors:** Zhen-Dong Sun, Yu-Ming Fang, Yan-Ling Lin, Meng-Qin Pei, Chu-Yun Liu, He-fan He

**Affiliations:** Department of Anesthesiology, The Second Affiliated Hospital of Fujian Medical University, Quanzhou, Fujian, China

**Keywords:** osteonecrosis of the femoral head, total hip arthroplasty, blood transfusion, machine learning, risk prediction, nomogram model

## Abstract

**Background:**

This study aimed to construct a predictive model utilizing multiple machine learning (ML) models to estimate the likelihood of perioperative blood transfusion in patients with osteonecrosis of the femoral head (ONFH) who underwent total hip arthroplasty (THA).

**Methods:**

Patients diagnosed with ONFH who underwent THA at our institution between October 2018 and October 2023 were included in the study. Feature selection was conducted using Lasso regression and correlation analysis. An unbiased evaluation framework incorporating nested resampling was established to assess four ML models. A nomogram was subsequently developed based on the selected features.

**Results:**

Seven features were identified, namely blood loss, hemoglobin (HGB) levels, weight, body temperature, systolic pressure, and direct bilirubin. Four ML models were constructed based on these features. The area under the curve (AUC) values for Random Forest, Extreme Gradient Boosting, Light Gradient Boosting Machine, and Logistic Regression (LR) were 1.00, 1.00, 1.00, and 0.93 in the internal validation set, and 0.89, 0.90, 0.88, and 0.91 in the external test set, respectively. Furthermore, a nomogram model based on LR was developed using the aforementioned seven features, yielding AUC values of 0.95 and 0.90 for the training and test sets, respectively, thereby surpassing the AUC values of preoperative HGB levels (0.80 and 0.76).

**Conclusion:**

Both the ML models and the nomogram exhibit significant potential for forecasting the likelihood of perioperative blood transfusion in patients with ONFH undergoing THA, which may aid clinicians in improving the accuracy of blood transfusion predictions.

## Introduction

Osteonecrosis of the femoral head (ONFH) is characterized by the necrosis of osteocytes resulting from ischemia of the femoral head (1). The global incidence is anticipated to reach 20 million within a decade (2), with an estimated 15,000–20,000 annual cases occurring in the United States (3). Currently, a definitive prevention method for the prevention for ONFH remains elusive, as non-surgical interventions apart from total hip arthroplasty (THA), are typically ineffective (4). However, THA has the potential to result in substantial blood loss, necessitating perioperative red blood cells (RBC) transfusions in up to 20% of patients (5, 6). Furthermore, allogeneic blood transfusions can lead to various adverse effects, including increased risks of surgical site infections, deep vein thrombosis, and mortality (7–9). Although some strategies can reduce the need for blood transfusions (10), these approaches may not be suitable for all patients due to associated risks (11).

Consequently, identifying patients who require perioperative blood transfusions is essential for adequate preparation and preventive measures. While physicians typically rely on hemoglobin (HGB) levels to inform transfusion decisions, it is crucial to also consider various perioperative factors such as age (12), sex (13), and laboratory values like alanine aminotransferase. Therefore, screening the variables that significantly influence blood transfusion and integrating them into a cohesive predictive model may enhance accuracy.

Machine learning (ML), a subset of artificial intelligence, employs computational modeling to extract insights from data. It improves the accuracy of predictions and classifications by utilizing large datasets for model training and continuously optimizing performance (14–16). ML has shown promise in clinical predictions across various medical specialties, including perioperative blood transfusion predictions (17, 18). However, there is limited scholarly research on the use of ML to predict perioperative transfusion requirements in patients with ONFH.

Thus, the objective of this research was to develop a predictive model to more accurately predict the likelihood of perioperative blood transfusion in patients with ONFH undergoing THA.

## Materials and methods

### Ethics statement and patient selection

This study adhered to the principles outlined in the Declaration of Helsinki and its amendments and received approval from the Ethics Committee at our hospital. The requirement for written informed consent was waived, and personal identifiers were anonymized prior to data analysis.

A retrospective analysis was conducted on a cohort of 503 patients with ONFH who underwent THA at our hospital between October 2018 and October 2023. The administration of packed RBC during hospital stays, referred to as perioperative blood transfusion, was determined through consultation between the anesthesiologist and the surgeon. Typically, a HGB concentration below 70 g/L indicates the necessity for a blood transfusion.

### Potential risk factors

We collected and evaluated the following features of the participants. General information: gender, age, the weight, height, and body mass index at the time of admission. Characteristics of surgery: operative site, surgery routes (anterior, posterior, and lateral approach), incisional length, intraoperative blood loss volume. Laboratory test results originated from the first post-admission blood draw: alanine aminotransferase [ALT], aspartate aminotransferase [AST], albumin [ALB], direct bilirubin, indirect bilirubin, international normalized ratio [NIR], prothrombin time [PT], activated partial thromboplastin time, fibrinogen, HGB levels, and platelet count. Others: vital signs at admission, intraoperative hemostatic drugs utility.

### Statistical analysis

Initially, we included 28 variables and had a sample size of 503, yielding a 15 EPV (Events per Predictor Variable), which surpasses the commonly recommended benchmark of 10 EPV (19, 20). Then, we standardized the continuous variables. The feature selection process utilized Lasso regression analysis with 10-fold cross-validation across the complete dataset. Features exhibiting pairwise correlation coefficients ≥0.6 (Pearson) were subsequently excluded through collinearity screening.

Following this, we extensively resampled non-blood transfusion samples to achieve a 1:1 ratio with blood transfusion samples. We then subjected four ML models—Random Forest (RF), Extreme Gradient Boosting (XGB), Light Gradient Boosting Machine (LGBM), and Logistic Regression (LR)—to hyperparameter tuning and performance evaluation via a nested sampling approach. In the inner loop, all parameter combinations were explored to identify optimal hyperparameters using random search, followed by model training. A four-fold cross-validation was employed for hyperparameter tuning, while a five-fold cross-validation was applied in the outer loop for model evaluation, ensuring the reliability and robustness of performance metrics. Consequently, the entire dataset was divided into training, internal validation, and external test sets in each fold. The evaluation of each ML model's performance was based on the average level of nested sampling. Subsequently, the performance of each model was assessed using receiver operating characteristic (ROC) curves, with a specific focus on the area under the curve (AUC) values. The DeLong test was utilized to verify statistical differences in AUC values among four ML models. Additionally, we employed various model performance evaluation metrics, including sensitivity, specificity, Brier scores, F-beta coefficient, positive predictive value (PPV), negative predictive value (NPV), Log Loss, and Matthews's correlation coefficient (MCC) to comprehensively assess model performance on both the internal validation set and the external test set.

Furthermore, bootstrap resampling was performed to partition the entire dataset into training and validation sets at a ratio of 503:186. Subsequently, a LR-based nomogram model was developed to predict the probability of perioperative blood transfusion based on the selected features. Model performance was rigorously evaluated using calibration curves, ROC curves, and decision curve analysis (DCA) in both the training and external validation sets. Statistical analyses were conducted using R software (version 4.4.1), with two-tailed tests applied throughout. Statistical significance was defined as P < 0.05.

## Results

### Clinical characteristics

In the entire dataset, notable differences in gender, weight, systolic pressure, diastolic pressure, operative site, blood loss, ALT, AST, ALB, indirect bilirubin, NIR, PT, and HGB levels were observed between patients in the transfusion and non-transfusion groups, with P below 0.05 as reported in Table 1. The remaining aggregate data did not exhibit statistically significant variances (*P* > 0.05).

**Table 1:** Baseline characteristics of patients in the study.

**Characteristic**	**Overall, *N* = 503**	**No transfusion, *N* = 418**	**Transfusion, *N* = 85**	***p-value***
**Gender**, ***n*** **(%)**				0.003
Male	276 (55)	242 (58)	34 (40)	
Female	227 (45)	176 (42)	51 (60)	
Age, Mean (SD)	56.58 (13.34)	56.63 (12.86)	56.33 (15.60)	0.81
Weight, Mean (SD)	63.03 (11.25)	63.57 (10.78)	60.36 (13.06)	0.006
Height, Mean (SD)	1.60 (0.12)	1.60 (0.12)	1.59 (0.12)	0.49
BMI, Mean (SD)	25.09 (5.73)	25.30 (5.83)	24.06 (5.13)	0.11
**Hypertension**, ***n*** **(%)**				0.38
No	384 (76)	316 (76)	68 (80)	
Yes	119 (24)	102 (24)	17 (20)	
**Diabetes**, ***n*** **(%)**				0.79
No	465 (92)	387 (93)	78 (92)	
Yes	38 (7.6)	31 (7.4)	7 (8.2)	
Body temperature, Mean (SD)	36.47 (0.19)	36.47 (0.19)	36.50 (0.20)	0.14
Respiratory rate, Mean (SD)	19.67 (1.12)	19.66 (1.03)	19.74 (1.51)	0.80
Pulse, Mean (SD)	81.18 (10.47)	81.32 (10.46)	80.48 (10.56)	0.60
Systolic pressure, Mean (SD)	131.10 (18.65)	132.03 (18.45)	126.56 (19.06)	0.020
Diastolic pressure, Mean (SD)	82.63 (10.91)	83.28 (10.91)	79.40 (10.40)	0.005
**Operative site**, ***n*** **(%)**				<0.001
Left	242 (48)	211 (50)	31 (36)	
Right	244 (49)	199 (48)	45 (53)	
Both	17 (3.4)	8 (1.9)	9 (11)	
**Surgery routes**, ***n*** **(%)**				0.60
Anterior	31 (6.2)	27 (6.5)	4 (4.7)	
Posterior	304 (60)	255 (61)	49 (58)	
External	168 (33)	136 (33)	32 (38)	
**Incisional length**, ***n*** **(%)**				0.060
<12	236 (47)	204 (49)	32 (38)	
≥12	267 (53)	214 (51)	53 (62)	
Blood loss, Mean (SD)	324.65 (292.19)	257.89 (132.38)	652.94 (540.36)	<0.001
**Hemostatic drugs**, ***n*** **(%)**				0.28
None	68 (14)	54 (13)	14 (16)	
Tranexamic acid	294 (58)	240 (57)	54 (64)	
Aminocaproic acid	134 (27)	118 (28)	16 (19)	
Aminomethylbenzoic acid	7 (1.4)	6 (1.4)	1 (1.2)	
ALT, Mean (SD)	21.94 (19.93)	22.20 (15.78)	20.64 (33.71)	<0.001
AST, Mean (SD)	22.95 (23.46)	23.21 (24.38)	21.70 (18.31)	0.007
Albumin, Mean (SD)	42.90 (4.85)	43.23 (4.77)	41.29 (4.90)	<0.001
Direct bilirubin, Mean (SD)	4.69 (10.38)	4.28 (1.93)	6.70 (24.90)	0.47
Indirect bilirubin, Mean (SD)	5.93 (3.04)	6.08 (3.07)	5.18 (2.73)	0.007
NIR, Mean (SD)	0.95 (0.07)	0.95 (0.07)	0.98 (0.09)	0.002
PT, Mean (SD)	11.83 (1.18)	11.77 (1.16)	12.17 (1.27)	0.008
APTT, Mean (SD)	28.41 (3.52)	28.35 (3.28)	28.70 (4.53)	0.19
FIB, Mean (SD)	3.64 (1.34)	3.62 (1.34)	3.74 (1.36)	0.58
HGB, Mean (SD)	133.87 (19.69)	137.26 (17.87)	117.19 (19.84)	<0.001
PLT, Mean (SD)	262.79 (78.09)	259.46 (71.72)	279.15 (102.88)	0.087

ALT, Alanine Aminotransferase; AST, Aspartate Aminotransferase; NIR, International Normalized Ratio; PT, Prothrombin Time; APTT, Activated Partial Thromboplastin Time; FIB, Fibrinogen; HGB, Hemoglobin; PLT, Platelet Count.

### Feature selection

**Figure 1:**
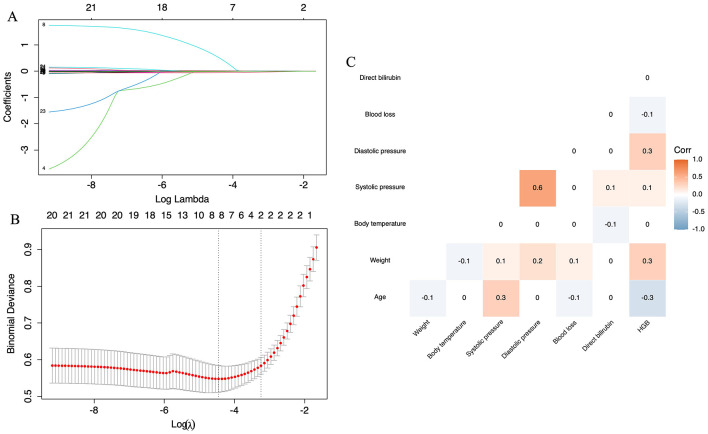
Lasso regression and Spearman's correlation analyses. **(A)** Distribution map illustrating the Lasso coefficients for all variables. **(B)** Identification of variables through Lasso regression analysis. **(C)** Correlation analysis conducted among the variables selected by Lasso regression.

To mitigate overfitting, a two-step feature selection process was implemented. Initially, Lasso Regression with 10-fold cross-validation was employed to identify eight features exhibiting stable associations (Figures 1A, B), namely blood loss, HGB levels, weight, body temperature, systolic pressure, diastolic pressure, and direct bilirubin. Subsequently, redundancy was minimized by calculating pairwise Spearman's correlations among these features and removing one variable from any pair with *r* ≥ 0.6, specifically excluding systolic blood pressure (Figure 1C). This strategy integrated regularized regression and correlation-based simplification to emphasize generalizable, non-redundant features. Binary classifications were constructed from original variables using clinically informed thresholds. Ultimately, seven variables—blood loss volume, HGB levels, weight, body temperature, systolic pressure, and direct bilirubin—were incorporated into the ML models.

### Development and validation of ML models

**Figure 2:**
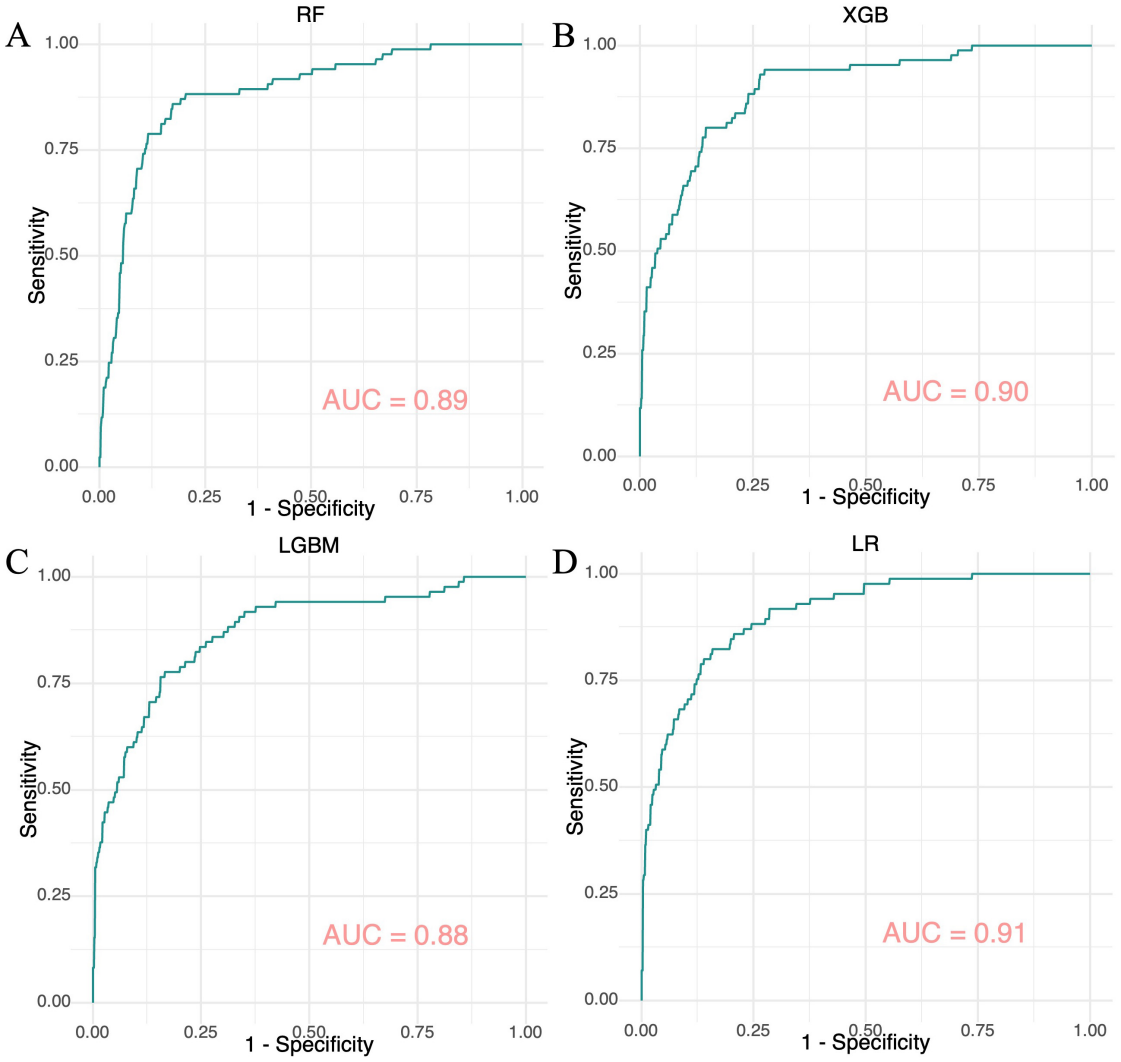
ROC curves for four machine learning models evaluated on the internal validation set. **(A)** The ROC curve for the Random Forest model. **(B)** The ROC curve for the Extreme Gradient Boosting model. **(C)** The ROC curve for the Light Gradient Boosting Machine model. **(D)** The ROC curve for the Logistic Regression model.

Using the seven selected features, four ML models were developed within a nested resampling framework to reduce the risk of overfitting. Results from systematic hyperparameter tuning for each model are summarized in Supplementary Table S1. The ROC curves for the RF, XGB, LGBM, and LR models in the external test set are illustrated in Figure 2, while those for the internal validation set are depicted in Supplementary Figure S1. AUC values for these models were 1.00, 1.00, 1.00, and 0.93 in the internal validation set (Supplementary Figure S1) and 0.89, 0.90, 0.88, and 0.91 in the external test set (Figure 2), respectively.

Furthermore, Tables 2, 3 present several key performance indicators for the models, including sensitivity, specificity, Brier score, F-beta coefficient, PPV, NPV, Log Loss, and MCC, indicating that most models demonstrated strong performance. The tree-based ensemble models (RF, XGB, and LGBM) achieved perfect training metrics; however, they exhibited significant declines in validation sensitivity and PPV, dropping to 0.59–0.62. LR achieved the highest external test AUC (0.91) with minimal sensitivity loss (0.80 compared to 0.81 in the external test set). Prediction uncertainty increased across all models during validation, with RF demonstrating the least reduction in performance.

**Table 2:** Performance evaluation of machine learning models within the internal validation set.

**Learner**	**AUC**	**Sensitivity**	**Specificity**	**Brier**	**F-beta**	**PPV**	**NPV**	**Log loss**	**MCC**
RF	1.00	1.00	0.99	0.05	0.98	0.97	1.00	0.14	0.98
Xgb	1.00	1.00	1.00	0.00	1.00	1.00	1.00	0.01	1.00
Lgb	1.00	1.00	1.00	0.00	1.00	1.00	1.00	0.00	1.00
LR	0.93	0.81	0.85	0.23	0.64	0.53	0.96	0.38	0.56

**Table 3:** Performance evaluation of machine learning models within the external test set.

**Learner**	**AUC**	**Sensitivity**	**Specificity**	**Brier**	**F-beta**	**PPV**	**NPV**	**Log loss**	**MCC**
RF	0.89	0.61	0.92	0.19	0.61	0.62	0.92	0.33	0.54
Xgb	0.90	0.60	0.92	0.21	0.59	0.59	0.92	0.39	0.51
Lgb	0.88	0.60	0.92	0.24	0.59	0.59	0.92	0.68	0.51
LR	0.91	0.80	0.85	0.25	0.64	0.53	0.95	0.40	0.56

**Figure 3:**
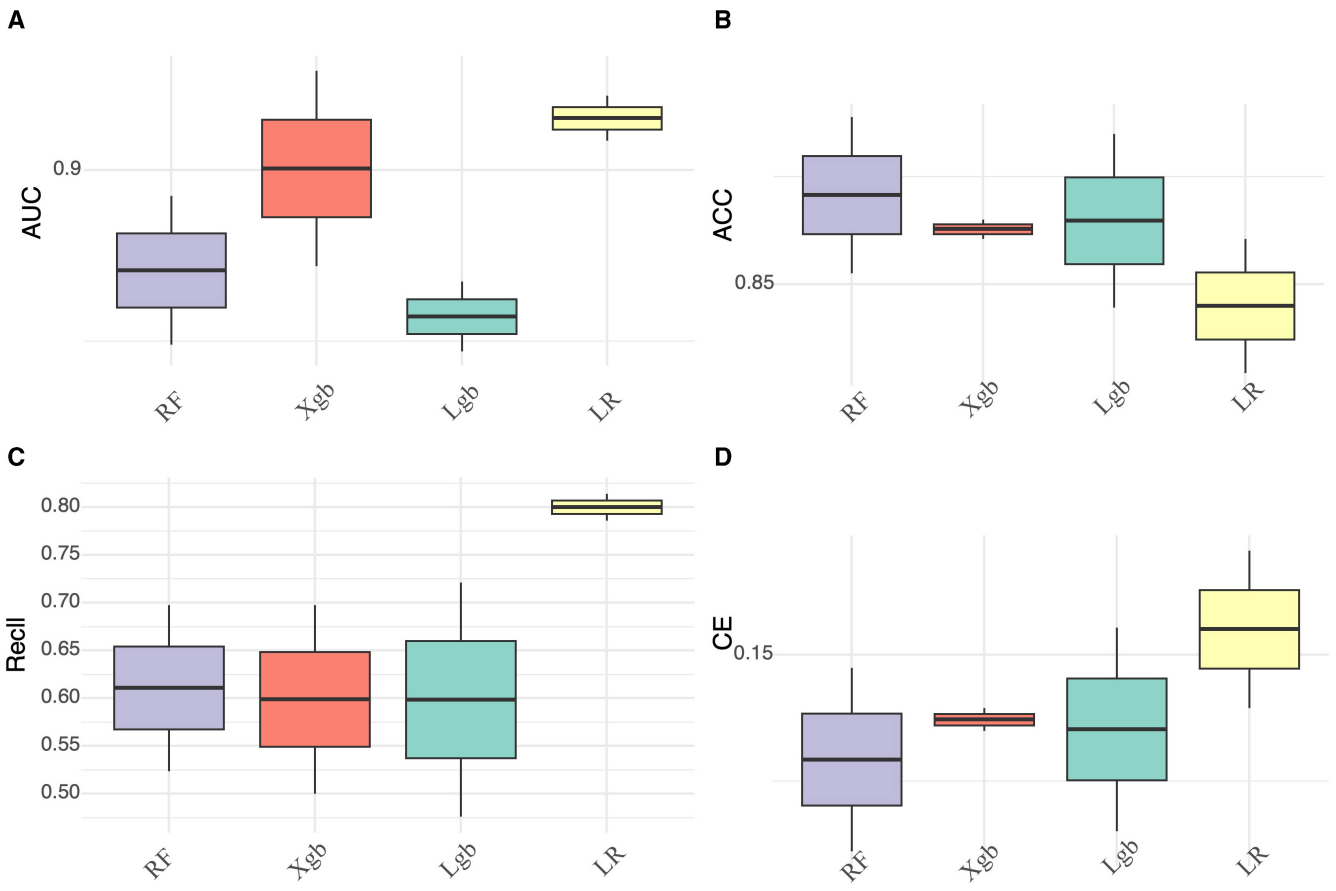
Box plot comparing four machine learning models on the external test set. **(A)** AUC values, **(B)** ACC values, **(C)** Recall values, and **(D)** CE values.

**Figure 4:**
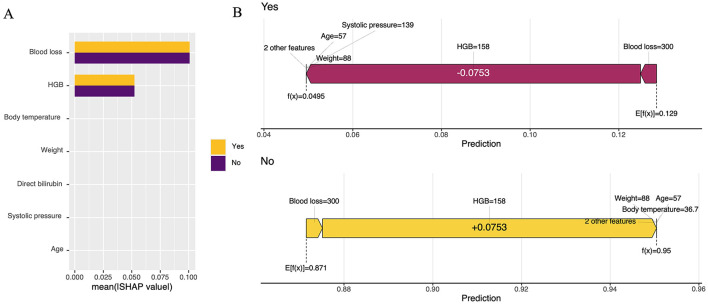
Statistical visualizations of the SHAP analysis. **(A)** An ordered plot illustrating the importance of variables in the SHAP analysis; **(B)** SHAP value contribution graph for a single sample's indicators.

Additionally, box plots provide an intuitive visualization of AUC, recall, ACC, and Cross-Entropy (CE) in relation to classification performance evaluation (Figure 3; Supplementary Figure S2). The confusion matrix for the four ML models is presented in Supplementary Figure S3, along with the DCA curves in Supplementary Figure S4. To investigate statistically significant differences in AUC values among the four ML models, we conducted the DeLong test (Supplementary Figure S5), which revealed no significant difference in AUC values between LR and the other ML models in external test set. Interpretation of the LR model was performed to reduce ML model complexity (Figure 4).

### Nomogram model construction and validation

**Figure 5:**
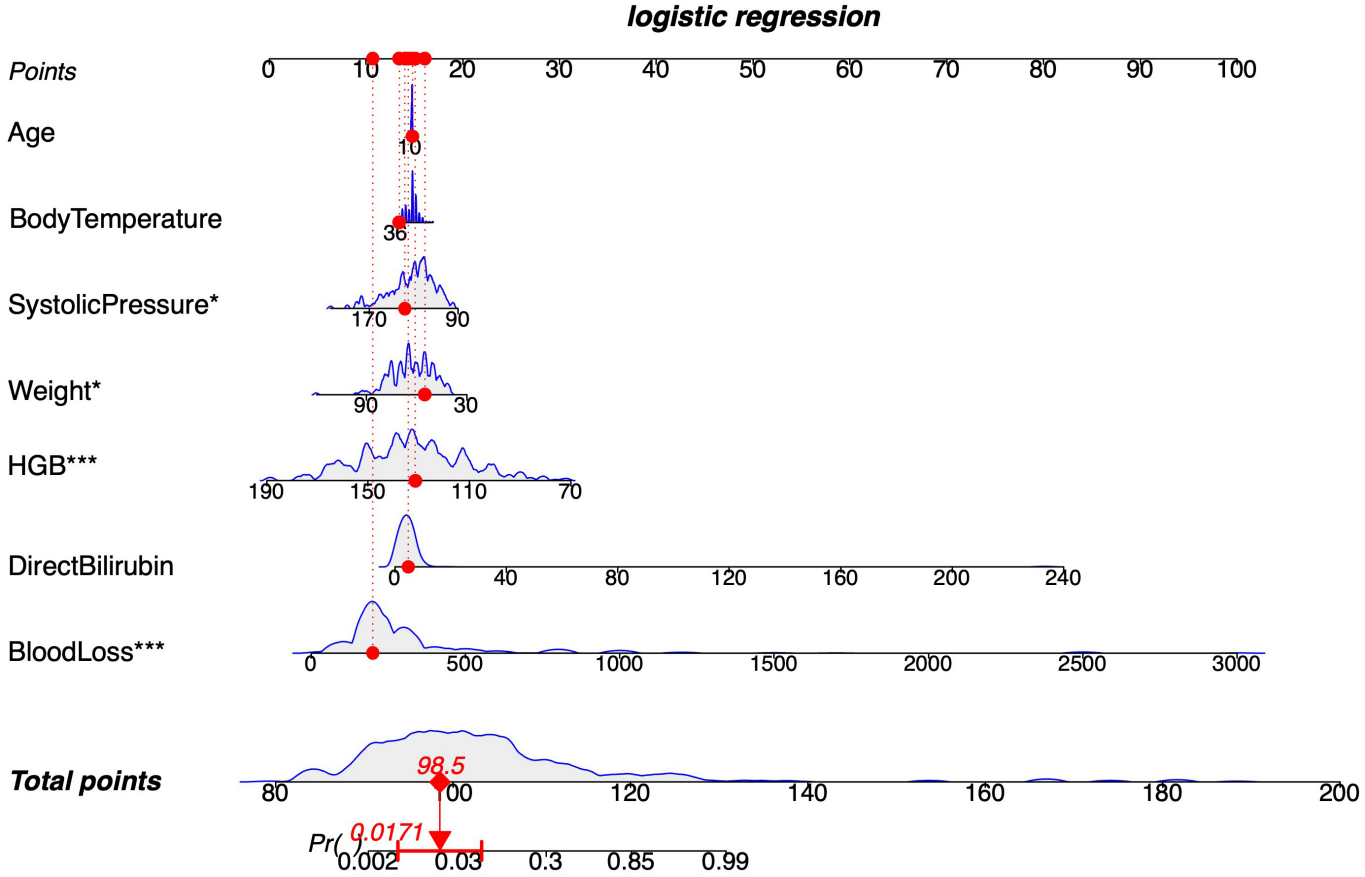
Nomogram model construction based seven features. ^*^Denotes the magnitude of the *P*-value.

**Figure 6:**
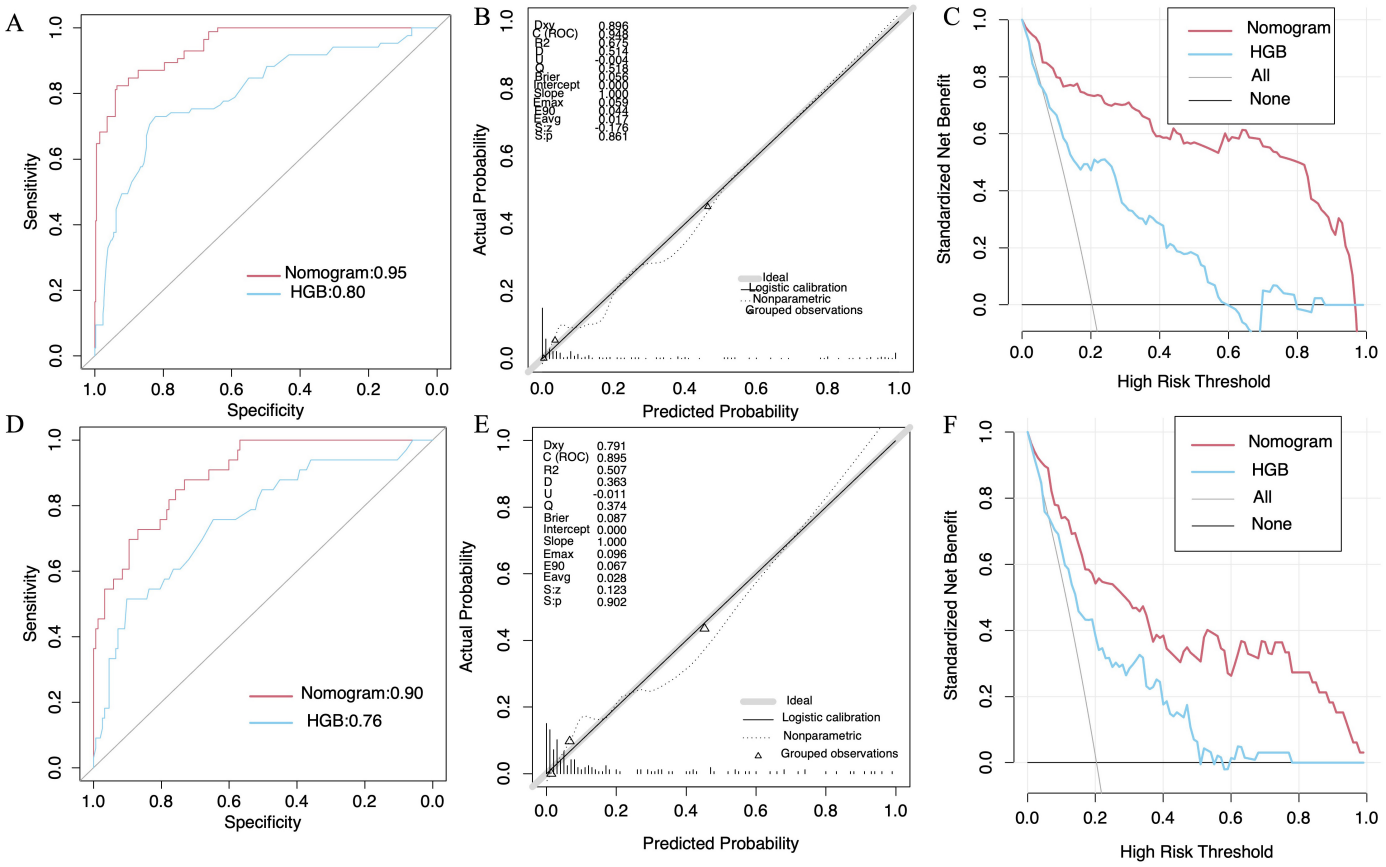
The receiver-operating characteristic curves, calibration plots, and decision curve analysis for the nomogram model in the training and validation sets are represented by **(A–F)**, respectively.

Moreover, to provide clinicians with a more effective visual assessment tool, a nomogram model incorporating the seven features was developed based on LR (Figure 5). Baseline data for the features are detailed in Supplementary Table S2. Calibration plots indicated strong alignment between expected and actual outcomes in both the training and validation sets, as evidenced by low Brier scores (0.056 for the training set and 0.087 for the validation set) (Figures 6B, E). To assess the clinical efficacy of the nomogram model, we conducted a direct comparison to evaluate its predictive capacity against the preoperative HGB levels of patients. The AUC values for the nomogram model were found to exceed those of the preoperative HGB levels in the training set (0.95, 95% CI = 0.93–0.97) and in the validation set (0.90, 95% CI = 0.84–0.95), compared to training set values of 0.80 (95% CI = 0.74–0.86) and 0.76 (95% CI = 0.66–0.85) in the validation set (Figures 6A, D). This demonstrates the superior discriminative capacity of the nomogram model. DCA curves further confirmed that the nomogram model offers greater net benefits in both cohorts (Figures 6C, F). Importantly, the nomogram model exhibited enhanced diagnostic performance compared to HGB levels, as indicated by the results of AUC and DCA analyses.

## Discussion

A prevalent complication associated with THA is considerable blood loss, frequently necessitating allogeneic blood transfusions. Such transfusions have been correlated with adverse outcomes and heightened healthcare costs (21), prompting clinicians to adopt strategies aimed at minimizing their necessity (22, 23). Predicting the need for perioperative blood transfusions can assist in identifying high-risk patients, improving patient care, and yielding better outcomes, lower morbidity rates, and cost savings. Consequently, anticipating transfusion needs and implementing appropriate preoperative interventions is essential.

Traditionally, physicians have relied on HGB levels to guide blood transfusion decisions (24, 25), however, significant discrepancies in the transfusion thresholds have been noted (26, 27). In this study, we identified seven key features—blood loss volume, HGB levels, body weight, body temperature, systolic pressure, and direct bilirubin—as predictive factors for the necessity of blood transfusions during the perioperative period.

The associations between the characteristic variables examined in this study and perioperative transfusions have been documented extensively in the literature. Previous studies have demonstrated a positive correlation between blood loss and the likelihood of requiring a blood transfusion (28, 29). Our findings align with these observations. Intraoperative blood loss may elevate the demand for blood transfusions, potentially due to a complex physiological cascade that includes reductions in blood volume, oxygenation, coagulation function, metabolic acidosis, impaired immune response, and other contributing factors.

HGB levels play a critical role in determining the necessity of perioperative blood transfusions (30, 31). One study revealed a five-fold increase in the likelihood of requiring a blood transfusion when preoperative HGB levels fall below 120 g/L (32), and another study arrived at a similar conclusion (33). This phenomenon can be attributed to the diminished ability of patients with lower preoperative HGB levels to effectively compensate for intraoperative blood loss (34).

Our results indicate that individuals with lower body weight are more likely to require a blood transfusion than those with higher body weight. Several studies have corroborated these findings (35, 36). Patients with lower body weight possess a reduced RBC volume (37, 38), which may hinder their ability to compensate for blood loss. Consequently, lighter individuals may find it more challenging to recover from significant blood loss compared to those with higher body weight (37). Additionally, studies have indicated that factors such as age, systolic pressure, and direct bilirubin significantly influence perioperative blood transfusions (9). Maintaining normal body temperature is crucial, as hypothermia can exacerbate blood loss and increase the need for transfusions. Research on THA demonstrates that stable body temperatures can mitigate blood loss and reduce transfusion rates (39).

ML has capabilities for effectively managing non-linear and complex data structures and addressing issues associated with high-dimensional data and missing values. Concurrently, it exhibits a robust capacity for pattern recognition and utilization. Various studies have confirmed the utility of ML in clinical diagnosis and prognosis (40, 41). Recently, there has been growing interest in applying ML to the field of blood transfusion (42, 43). Currently, no models exist to predict the likelihood of perioperative blood transfusions in patients with ONFH undergoing THA. While some predictive models perform well, they face clinical application challenges due to suboptimal performance (44). Moreover, there is a notable gap in evaluating predictive performance specific to ONFH cases.

In this study, we developed four ML models through hyperparameter tuning for perioperative blood transfusions in patients with ONFH undergoing THA. RF mitigates the risk of overfitting by integrating multiple decision trees and demonstrates exceptional robustness in the presence of high-dimensional data and noise (45). Furthermore, it facilitates the assessment of feature importance (46). XGB employs second-order Taylor expansion and incorporates regularization terms, resulting in enhanced prediction accuracy and robust generalization (47). The LGBM utilizes histogram algorithms and prioritizes the growth of leaf nodes, achieving remarkably fast training speeds and low memory consumption, which renders it suitable for ultra-large-scale datasets (47). LR offers strong interpretability of probability outputs, allows for the adjustment of decision thresholds, and is characterized by its simplicity, computational efficiency, and speed, making it suitable for large-scale structured data (48). LR was deemed the most clinically suitable due to its balanced performance across various metrics. The RF, XGB, and LGBM models exhibited excellent results but had low Brier scores and Log Loss, indicating overfitting to the internal validation set. Their performance significantly declined on the external test set, particularly regarding sensitivity and PPV, highlighting their limited generalizability. Additionally, the AUC values of the four models did not display any statistical differences in DeLong test conducted on the external test set. LR demonstrated clinical validity through four primary features in our study: first, it possessed higher validation sensitivity, ensuring better identification of true transfusion candidates, which is critical in surgical settings. Second, it balanced specificity and NPV, maintaining strong negative classification without missing positive cases. Third, it exhibited stable performance from internal validation to external test, showing the smallest AUC decrease, indicating reliable generalization. Finally, the DCA illustrated that the LR model yields a significant net benefit and maintains consistency between the internal validation and external test sets. Ensemble methods displayed slightly better calibration and Log Loss; however, their reduced sensitivity renders them clinically less viable. The higher MCC of LR indicates its superior classification accuracy in clinical settings.

The SHapley Additive exPlanations (SHAP) is a method for interpreting ML models that is grounded in the Shapley value from game theory (49). By quantifying the marginal contribution of each feature to the model's predictions, SHAP offers both global and local interpretability (50). The SHAP analysis identified critical features: HGB levels (positive correlation), body weight (negative correlation), and age (strong global influence) (9). High HGB levels suggest compensatory hemoconcentration during blood loss, while greater body weight indicates a better physiological reserve (37). Advanced age is strongly correlated with risk due to diminished hemodynamic adaptability. Blood loss and hypothermia exhibited synergistic effects, correlating with clinical observations of worsened coagulopathy HGB (29). These patterns confirm the model's alignment with established pathophysiology, underscoring its clinical relevance.

We also constructed a nomogram based on LR to predict transfusion risk. The nomogram demonstrated robust discriminatory ability, with consistent AUC values in both training and validation sets. Direct comparisons revealed that the predictive performance of this nomogram significantly outperformed HGB levels. Furthermore, the DeLong test indicated a statistically significant difference in AUC values between the nomogram and both LR and HGB levels. This finding demonstrates that the predictive capabilities of LR and the nomogram exceed those of HGB levels. Moreover, the DCA curves indicate that the nomogram model provides a substantially higher net benefit compared to HGB levels. This visual tool supports clinicians in risk assessment, allowing for improved perioperative blood management and surgical planning. By identifying high-risk patients, it may help reduce transfusion-related complications through targeted interventions. The nomogram's combination of clarity and accuracy makes it an invaluable tool for enhancing perioperative care in neurovascular surgeries.

This model aids in identifying patients requiring perioperative blood transfusions and optimizing blood management strategies, such as autologous blood dilution, intraoperative hypotension management, and postoperative recovery. For successful integration into clinical practice, it is vital to clearly define the model's objectives, emphasizing the enhancement of predictive accuracy to prevent unnecessary transfusions. The clinical implementation plan consists of four key components: (1) Electronic Medical Record (EMR) integration via HL7 interfaces to automatically extract data from laboratories and operative notes; (2) Real-time prediction modules capable of issuing prioritized alerts during surgical scheduling based on risk; (3) Multidisciplinary protocols to monitor transfusion deviations and response times at three pilot sites; and (4) Oversight by a blood management committee that recalibrates models biannually using federated learning across institutions. This framework ensures clinical utility, regulatory compliance, and auditability through performance dashboards.

This study has certain limitations, including single-center data sourcing and the potential for selection bias. The retrospective single-center design inherently restricts the external validation of these findings. Moreover, retrospective studies inevitably involve missing data. Although rigorous internal controls were employed, the absence of multi-institutional data may limit the generalizability of results to broader populations. Furthermore, the retrospective nature of this study necessitates careful consideration of potential confounding factors. But factors such as prior surgical interventions, comorbidities beyond hypertension and diabetes, and smoking were not considered in the present study. We plan to incorporate these variables into our model in future research. Additionally, the retrospective design prevents precise estimations of clinical decision thresholds; as such analyses require outcome data from actual implementation scenarios. Future work will encompass cost-benefit analysis with stakeholder engagement to operationalize these thresholds. Research directions include multicenter validation, prospective studies, exploration of new data sources and features, and continuous iteration and optimization of the model to enhance its generalizability, accuracy, and clinical applicability. Furthermore, our models have only undergone internal validation and require external validation to confirm their effectiveness.

In conclusion, this study developed four ML models and a nomogram model to effectively predict the likelihood of blood transfusion in patients with ONFH undergoing THA. The model's capability to identify patients with a low probability of requiring a transfusion could diminish unnecessary repeat testing, such as complete blood counts and additional preoperative laboratory tests. Additionally, it can assist clinicians in implementing strategies to reduce bleeding and prepare for transfusions in high-risk patients. Consequently, this model could serve as a valuable tool for clinicians in preoperative preparation and in reducing unnecessary medical procedures.

## Data Availability

The raw data supporting the conclusions of this article will be made available by the authors, without undue reservation.
