# Supplementary material for: Construction and validation of a perioperative blood transfusion model for patients undergoing total hip arthroplasty with osteonecrosis of the femoral head based on machine learning
Source: Front Med (Lausanne). 2025 Sep 11;12:1471746. doi: 10.3389/fmed.2025.1471746 (PMC12460462; doi:10.3389/fmed.2025.1471746)
Supplement: Supplementary file 1 [file Data_Sheet_1.pdf]

Supplemental Information

Construction and validation of a perioperative blood transfusion model for patients undergoing total hip arthroplasty with osteonecrosis of the femoral head based on machine learning

Figure S1-S6
Table S1-S2

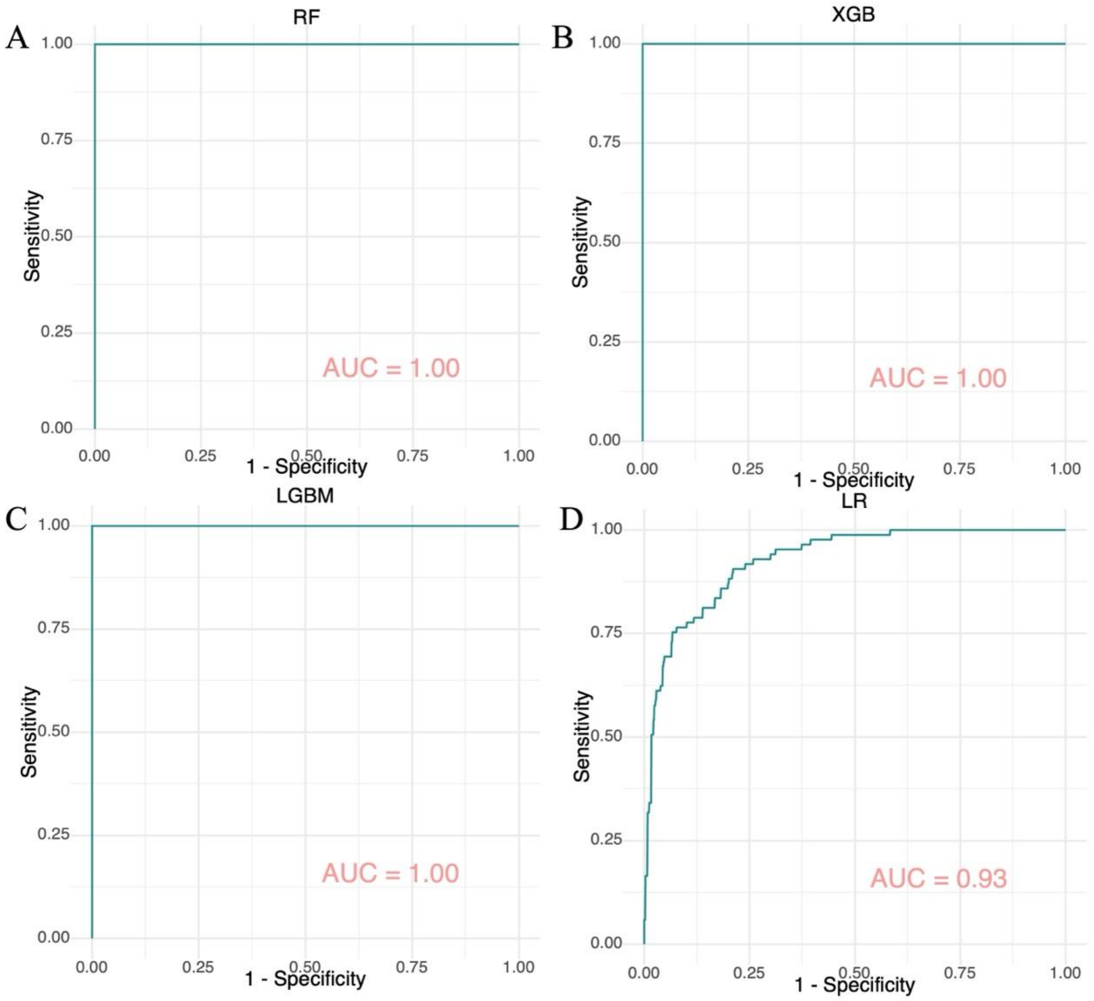

Figure S1. ROC curves for four machine learning models evaluated on the internal validation set. (A) The ROC curve for the Random Forest model indicates a value of 1.00. (B) The ROC curve for the Extreme Gradient Boosting model indicates a value of 1.00. (C) The ROC curve for the Light Gradient Boosting Machine model indicates a value of 1.00. (D) The ROC curve for the Logistic Regression model indicates a value of 0.93.

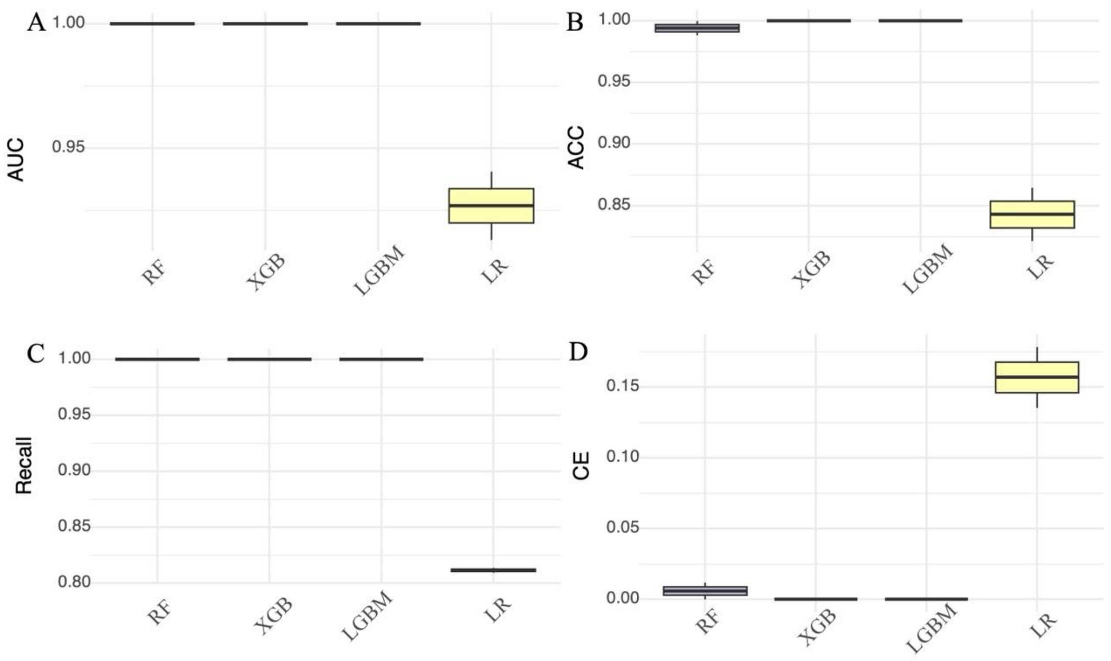

Figure S2. Box plot comparing four machine learning models on the internal validation set: (A) AUC values, (B) ACC values, (C) Recall values, and (D) CE values.

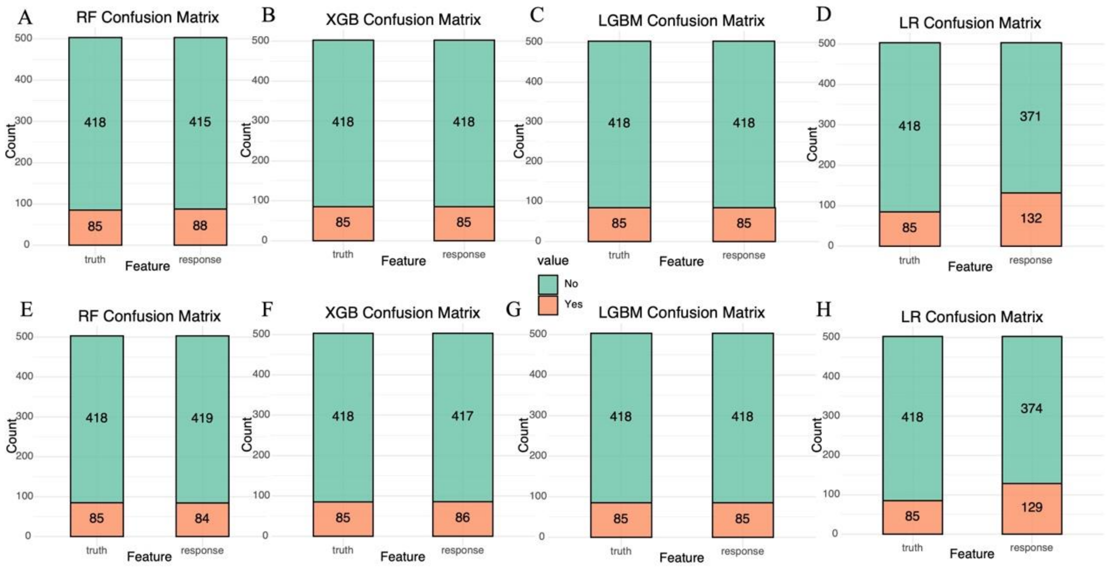

Figure S3. Confusion Matrix. (A) Random Forest (RF) confusion matrix, (B) Extreme Gradient Boosting (XGB) confusion matrix, (C) Light Gradient Boosting Machine (LGBM) confusion matrix, and (D) Logistic Regression (LR) confusion matrix within the internal validation set; (E) Random Forest (RF) confusion matrix, (F) Extreme Gradient Boosting (XGB) confusion matrix, (G) Light Gradient Boosting Machine (LGBM) confusion matrix, and (H) Logistic Regression (LR) confusion matrix within the external test set.

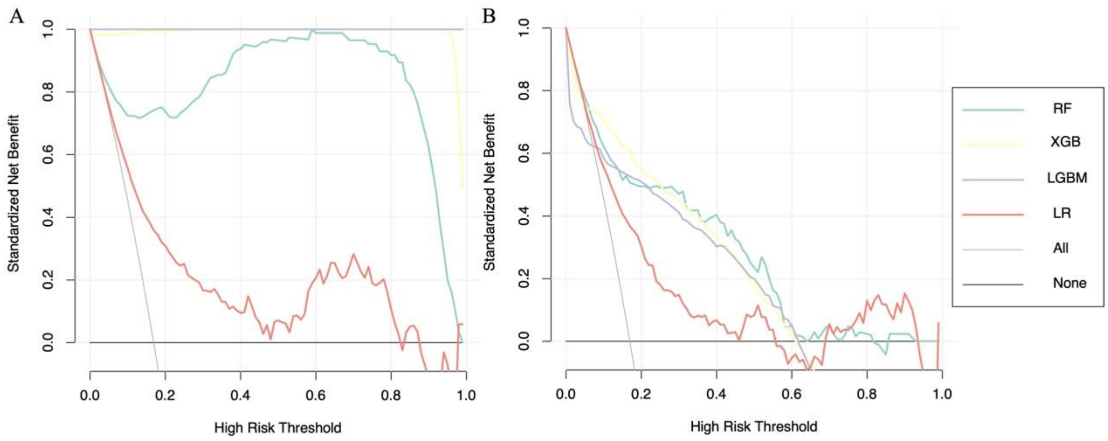

Figure S4. Decision Curve Analysis. (A) The Decision Curve Analysis (DCA) curves for four machine learning models within the internal validation set. (B) The DCA curves for the same four machine learning models within the external test set.

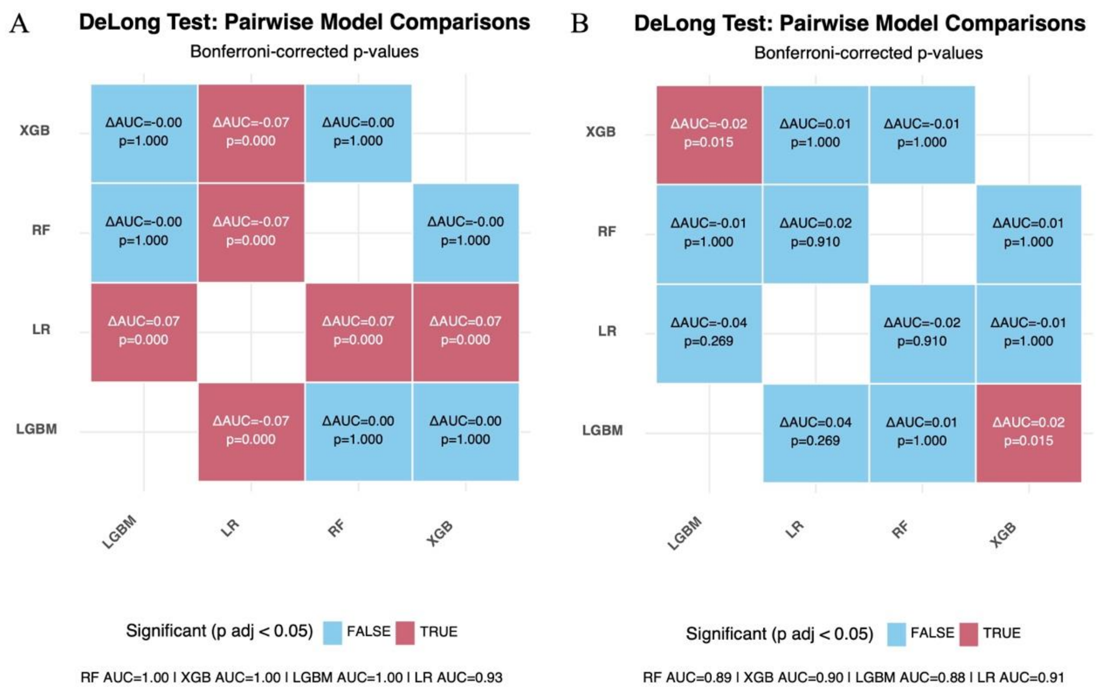

Figure S5. Delong test for four machine learning models. (A) within the internal validation set. (B) within the external test set.

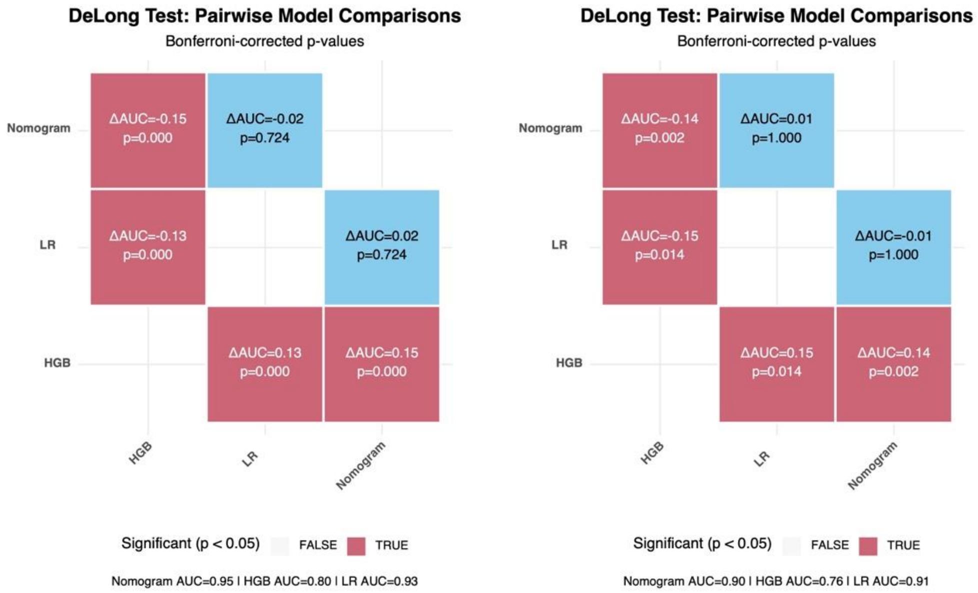

Figure S6. Delong test for four nomogram, hemoglobin and Logistic Regression. (A) within the internal validation set. (B) within the external test set.

Table S1. Parameters used in machine learning models

**Parameters used in machine learning models**	
**RF**	
Min node size	23
Mtry	1
Num trees	130
**XGB**	
eta	0.08675
Max depth	3
nrounds	90
subsample	0.6389
**LGBM**	
Learning rate	0.08311
Num leaves	53
Max depth	8
Min data in leaf	44
Num iterations	170
**LR**	
Alpha	0.3678
Nlambda	100

Table S2. Baseline characteristics of patients in bootstrap resampling.

Characteristic	Overall<br>N = 689	training<br>N = 503	validation<br>N = 186	p-value[1]
Transfusion, n (%)				0.79
No	571 (83)	418 (83)	153 (82)	
Yes	118 (17)	85 (17)	33 (18)	
Age, Mean (SD)	56.63 (13.44)	56.81 (13.54)	56.13 (13.20)	0.55
Weight, Mean (SD)	62.83 (11.70)	62.84 (12.23)	62.78 (10.17)	0.84
Body temperature, Mean (SD)	36.47 (0.19)	36.48 (0.19)	36.46 (0.20)	0.16
Systolic pressure, Mean (SD)	131.56 (19.19)	131.73 (19.91)	131.09 (17.13)	0.95
Blood loss, Mean (SD)	324.98 (309.72)	326.62 (334.67)	320.54 (229.75)	0.31
Direct bilirubin, Mean (SD)	5.61 (17.49)	6.11 (20.42)	4.27 (1.97)	0.56
HGB, Mean (SD)	133.19 (20.22)	132.72 (20.64)	134.47 (19.02)	0.31

[1]Pearson's Chi-squared test; Wilcoxon rank sum test
